# Modulation of IL-6 Expression by KLF4-Mediated Transactivation and PCAF-Mediated Acetylation in Sublytic C5b-9-Induced Rat Glomerular Mesangial Cells

**DOI:** 10.3389/fimmu.2021.779667

**Published:** 2022-01-03

**Authors:** Lu Xia, Yu Liu, Zhiwei Zhang, Yajuan Gong, Tianyi Yu, Dan Zhao, Wen Qiu, Yingwei Wang, Jing Zhang

**Affiliations:** ^1^ Department of Immunology, Nanjing Medical University, Nanjing, China; ^2^ Department of Microbiology and Immunology, Jiangsu Health Vocational College, Nanjing, China; ^3^ Key Laboratory of Immunological Environment and Disease, Nanjing Medical University, Nanjing, China; ^4^ Key Laboratory of Antibody Technology of Ministry of Health, Nanjing Medical University, Nanjing, China

**Keywords:** acetylation, KLF4, PCAF, sublytic C5b-9, IL-6

## Abstract

Interleukin-6 (IL-6) overproduction has been considered to contribute to inflammatory damage of glomerular mesangial cells (GMCs) in human mesangial proliferative glomerulonephritis (MsPGN) and its rat model called Thy-1 nephritis (Thy-1N). However, the regulatory mechanisms of IL-6 expression in GMCs upon sublytic C5b-9 timulation remain poorly understood. We found that Krüppel-like factor 4 (KLF4) bound to the IL-6 promoter (−618 to −126 nt) and activated IL-6 gene transcription. Furthermore, lysine residue 224 of KLF4 was acetylated by p300/CBP-associated factor (PCAF), which was important for KLF4-mediated transactivation. Moreover, lysine residue 5 on histone H2B and lysine residue 9 on histone H3 at the IL-6 promoter were also acetylated by PCAF, which resulted in an increase in IL-6 transcription. Besides, NF-κB activation promoted IL-6 expression by elevating the expression of PCAF. Overall, these findings suggest that sublytic C5b-9-induced the expression of IL-6 involves KLF4-mediated transactivation, PCAF-mediated acetylation of KLF4 and histones, and NF-κB activation in GMCs.

## Introduction

Renal inflammation, glomerular mesangial cell (GMC) proliferation, and matrix overproduction are key features of human mesangial proliferative glomerulonephritis (MsPGN), such as IgA nephropathy (1–3). Thy-1 nephritis (Thy-1N) as a popular experimental model for MsPGN is induced in the rat by injection of antibody to Thy-1, triggering activation of the complement system and subsequent formation of C5b-9 on the surface of GMC membranes (4–6). Previous studies have demonstrated that renal pathologic changes in Thy-1N rats are complement-dependent, especially sublytic C5b-9-dependent (7–10). In the early stage of Thy-1N, a number of proinflammatory cytokines are produced by GMCs in response to sublytic C5b-9 stimulus (8–10).

Of these proinflammatory cytokines, interleukin-6 (IL-6) overexpression has been detected in kidney biopsies and in the urine of patients with IgA nephropathy (11). Reportedly, GMCs can secrete IL-6, which plays an essential role in the immune-mediated injury of the kidney (11–14). For example, IL-6 promotes GMCs to synthesize and release MCP-1 and subsequently enhances monocyte recruitment, aggravating glomerular inflammation (13). IL-6 also induces matrix protein transcription and autocrine growth in GMCs (14). Although the exact role of IL-6 in MsPGN remains to be determined, exploring regulatory mechanisms of IL-6 expression in sublytic C5b-9-attacked rat GMCs is beneficial to understand the role of IL-6 in human MsPGN.

At the level of transcriptional regulation, transcription factors can interact with the IL-6 promoter to initiate its mRNA synthesis (15, 16). Krüppel-like factor 4 (KLF4) is an evolutionarily conserved zinc finger-containing transcription factor that regulates promoter activation *via* binding to GC box or CACCC element (17–20). It has been demonstrated that KLF4 modulates IL-6 production in dendritic cells (19) and fibroblast-like synoviocytes (20) *via* direct transcriptional activation of the IL-6 promoter. Our former experiments have found that IL-23 and IL-36a genes are regulated by KLF4 in GMCs (9). Therefore, in the present study, we sought to elucidate whether KLF4 also contributes to the transcriptional regulation of IL-6 in sublytic C5b-9-stimulated GMCs.

In addition to the binding of transcription factors to the IL-6 promoter, chromatin structure also plays a key role in regulating gene expression (21, 22). Histone acetylation, which is catalyzed by histone acetyltransferases (HATs) and is one determinant of transcription efficiency, is associated with an enhancement of access of transcription factors, leading ultimately to a more active state partly due to a weakened interaction between histone proteins and DNA strands (23, 24). P300/CBP-associated factor (PCAF), a HAT, acetylates histone H3 (25, 26) or non-histone proteins such as transcription factors (9, 27) and regulates inflammatory molecules in the progression of renal injury (25). Acetylation of transcription factors has diverse effects on transcriptional activity, including their DNA binding activity, interactions with other transcriptional regulators, and nucleocytoplasmic shuttling (28). Our previous study has demonstrated that KLF4 is acetylated by PCAF, which has a positive role in the transcriptional regulation of IL-23 and IL-36a (9). However, the role of PCAF-mediated acetylation of histones and KLF4 in the regulation of IL-6 expression in sublytic C5b-9-triggered GMCs is still unclear.

In brief, the aim of this study is to explore whether KLF4 and PCAF can regulate IL-6 production by rat GMCs in response to sublytic C5b-9 attacks. The results found that KLF4 bound to the IL-6 promoter and activated its transcription. Moreover, PCAF acetylated KLF4 in addition to acetylating neighboring histones H2B and H3 and resulted in an increase of IL-6 transcription. Besides, NF-κB activation promoted IL-6 expression by elevating PCAF expression rather than by changing KLF4 expression.

## Materials and Methods

### Animals, Cell Line, and Reagents

Male Sprague–Dawley rats (180–200 g) were purchased from the Animal Core Facility of Nanjing Medical University. All animal experiments were performed in compliance with the Guide for the Care and Use of Laboratory Animals and were approved by the Institutional Animal Care and Use Committee of Nanjing Medical University. Rat GMC strain (HBZY-1) was provided by China Center for Type Culture Collection (Wuhan, China).

Rabbit polyclonal anti-Thy-1 antibody (Ab, 1:640) and normal rabbit serum (NS) were prepared in our laboratory according to the previous document (8–10). Normal human serum (NHS) pooled from 30 healthy adult donors was used to supply complement. Heat-inactivated serum (HIS) was obtained by incubating NHS at 56°C for 30 min. Human C6-deficient serum (C6DS) was from Sigma-Aldrich (USA). Recombinant human C6 was from Sino Biological (China). Abs against KLF4 (sc-20691X) and histone H3 (sc-10809) were purchased from Santa Cruz (USA). Ab against IL-6 (A0286) was from ABclonal Technology (China). Abs against NF-κB p65 (abs131170) and phospho-NF-κB p65 (abs130624) were supplied by Absin Bioscience Inc. (China). Abs against histone H2B (ab52599), histone H2B acetyl K5 (ab40886), histone H2B acetyl K15 (ab62335), histone H2B acetyl K20 (ab52988), histone H3 acetyl K9 (ab32129), histone H3 acetyl K14 (ab52946), histone H3 acetyl K18 (ab40888), and PCAF (ab176316) were from Abcam (USA). Ab against β-actin (AF0003) and IgG (A7016) were supplied by Beyotime (China). Inhibitors, including BP-1-102 (S7769), SP600125 (S1460), LY294002 (S1105), perifosine (S1037), Torin 1 (S2827), SB203580 (S1076), BAY 11-7082 (S2913), and rapamycin (S1039), were purchased from Selleck (USA). Anacardic acid (16611-84-0) was from Santa Cruz (USA).

### Rat Thy-1 Nephritis Model Establishment

Sprague–Dawley rats were divided into two groups (n = 6 in each time point): Thy-1N group rats were injected intravenously with Thy-1 Ab (1 ml/100 g) by intravenous injection; NS group rats were injected intravenously with normal rabbit serum (1 ml/100 g). Renal cortex samples were obtained by sacrifice at different times.

### Cell Culture, Sublytic C5b-9 Stimulation, and Inhibitor Pretreatment

GMCs were cultured in minimum essential medium (MEM; Gibco, USA) containing 10% fetal bovine serum (FBS; Wisent, Canada). Lactate dehydrogenase (LDH) was detected in the supernatants of cultured cells, and <5% LDH release from cells was regarded as sublytic (8). According to this criterion, 5% Thy-1 Ab plus 4% NHS was used to assemble sublytic C5b-9 on GMC membrane as previously described (8). GMCs were also treated into other groups: Thy-1 Ab group represented GMCs incubated only with 5% Thy-1 Ab; Thy-1 Ab + HIS group represented 5% Thy-1 Ab-sensitized GMCs incubated with 4% HIS; Thy-1 Ab + C6DS group represented 5% Thy-1 Ab-sensitized GMCs incubated with 4% C6DS; and Thy-1 Ab + C6DS + C6 group represented 5% Thy-1 Ab-sensitized GMCs incubated with 4% C6DS and then reconstituted with 2 mg/L of C6.

In some experiments, GMCs were pretreated with inhibitors including BP-1-102 (10 μM), SP600125 (10 μM), LY294002 (10 μM), perifosine (10 μM), Torin 1 (10 μM), SB203580 (10 μM), BAY 11-7082 (10 μM), and rapamycin (10 μM) for 30 min, followed by sublytic C5b-9 stimulation for 3 h. In some other experiments, GMCs were pretreated with anacardic acid (30 μM) for 1 h and then stimulated with sublytic C5b-9 for 3 h.

### Plasmids and Cellular Transfection

All plasmids were constructed in our laboratory according to the previous documents (9). The following constructions were used: the overexpression plasmids such as pIRES2-KLF4 and pIRES2-PCAF; the short hairpin RNA (shRNA) plasmids such as shKLF4 and shPCAF; the scrambled control shRNA (shCTR) expression plasmid as a negative control; deleted HAT domain mutant such as PCAFΔHAT; lysine residue mutants such as KLF4-K224R and KLF4-K224Q; the luciferase reporter vectors of the IL-6 promoter such as full-length (FL), −1,392 to +30, −890 to +30, −618 to +30, and −126 to +30 nt.

GMCs were transfected with the corresponding plasmids using the Neon™ Transfection System (Invitrogen, USA). A total of 4 × 10^5^ cells were resuspended in 100 μl of resuspension buffers, including 3 μg of plasmids, and electroporated at 1,600 V (20 ms, 1 time). The cells were then transferred to a six-well plate.

### Quantitative PCR and Reverse Transcription PCR Assays

Total RNA was extracted from rat renal cortex or GMCs using TRIzol reagent (Invitrogen, USA) and reverse-transcribed into cDNA using HiScript 1st Strand cDNA Synthesis Kit (Vazyme, China), according to the manufacturer’s protocol. The qPCR assay was performed using AceQ qPCR SYBR Green Master Mix (Vazyme, China), and RT-PCR assay was performed using 2× Taq Master Mix (Vazyme, China). Primer sequences are shown in **Table 1**. Data were normalized to the β-actin expression.

**Table 1:** Specific primers used in PCR analysis.

Name	Primer	Sequence, 5′→3′
KLF4	FW	TGAACTGACCAGGCACTACC
	RV	GCCTCTTCATGTGTAAGGCA
IL-6	FW	ACTTCACAAGTCGGAGGCTT
	RV	AGTGCATCATCGCTGTTCAT
β-Actin	FW	TCACCCACACTGTGCCCATCTATGA
	RV	CATCGGAACCGCTCATTGCCGATAG
IL-6 (ChIP)	FW	GTGTGCGCACATGTGTTT
	RV	TAGCATCCAAAGAATCACAGC

FW, forward; RV, reverse; ChIP, chromatin immunoprecipitation.

### Immunoblotting Analysis

Renal cortex or GMC lysates were prepared in lysis buffer (Cell Signaling Technology, USA). Equal amounts of proteins were subjected to sodium dodecyl sulfate–polyacrylamide gel electrophoresis (SDS-PAGE). Immunoblotting (IB) analysis was performed as previously reported (8). β-Actin was used as an internal control for protein loading, and the relative protein level in each group was calculated by comparison with that in the control group.

### Enzyme-Linked Immunosorbent Assay

The supernatants from GMCs were collected for measurement of IL-6 using an ELISA kit (Shanghai Enzyme-linked Biotechnology Co., China) according to the manufacturer’s recommendations.

### Luciferase Reporter Assay

The FL or deletion promoter report of IL-6 was transiently transfected into GMCs with KLF4 or PCAF associated plasmids and then stimulated with or without sublytic C5b-9. Cell extracts were prepared, and luciferase activity was measured using the Dual-Luciferase Reporter Assay System (Promega, USA) as previously reported (8).

### Chromatin Immunoprecipitation Assay

GMCs were cross-linked with 1% formaldehyde and subjected to chromatin immunoprecipitation (ChIP) assay following the instructions of the Chromatin Immunoprecipitation Assay Kit (Millipore, USA). Purified DNA was analyzed by RT-PCR and qPCR, and the primers are listed in **Table 1**. Data were normalized to input DNA.

### Statistical Analysis

All experiments were performed in triplicate. Data are presented as mean ± SD. Statistical significance, defined as p < 0.05, was evaluated using one-way ANOVA or Student’s t-test.

## Results

### Expression of KLF4 and IL-6 Is Increased Both in Renal Tissue of Thy-1 Nephritis Rats and in Glomerular Mesangial Cell Stimulated With Sublytic C5b-9

**Figure 1:**
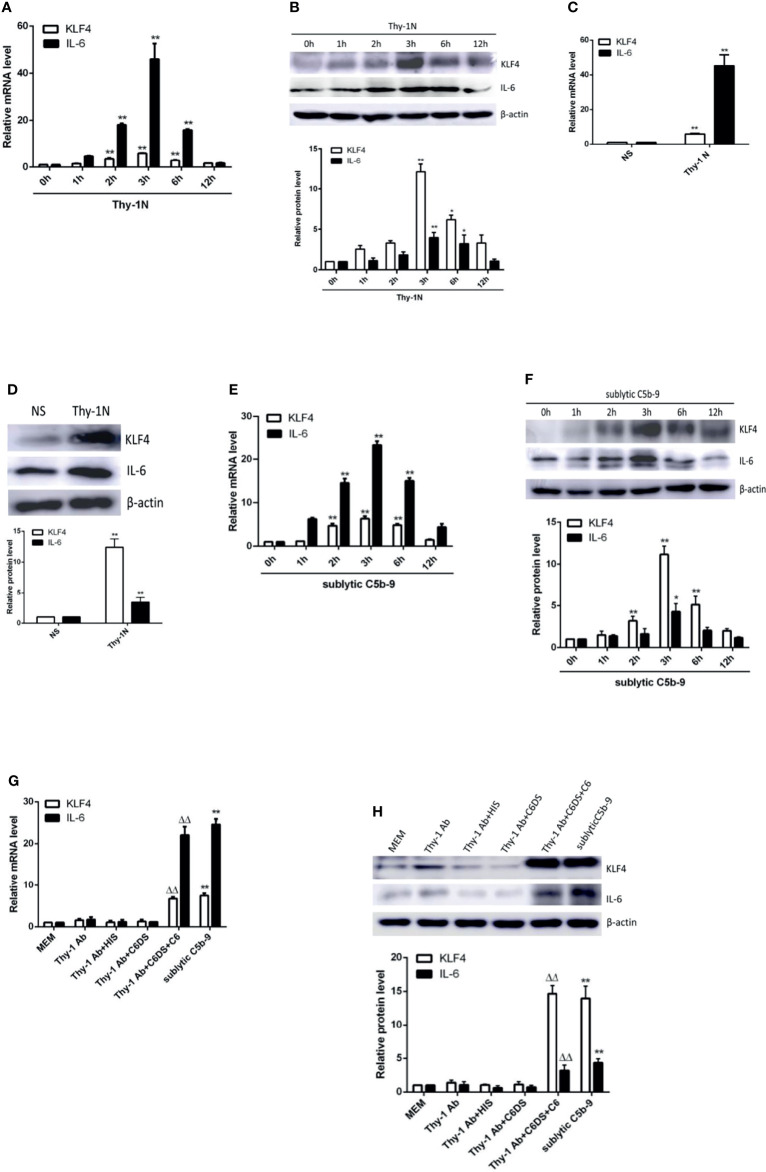
Expression of KLF4 and IL-6 both in renal tissue of Thy-1N rats and in GMCs stimulated by sublytic C5b-9. **(A**, **B)** qPCR and IB analyses of KLF4 and IL-6 in renal cortex of Thy-1N rats at indicated times (*p < 0.05, **p < 0.01 vs. 0 h). **(C**, **D)** qPCR and IB analyses of KLF4 and IL-6 in renal cortex of Thy-1N and NS rats for 3 h (**p < 0.01 vs. NS). **(E**, **F)** qPCR and IB analyses of KLF4 and IL-6 in GMCs treated with sublytic C5b-9 at indicated times (*p < 0.05, **p < 0.01 vs. 0 h). **(G**, **H)** qPCR and IB analyses of KLF4 and IL-6 in GMCs with different treatments for 3 h (**p < 0.01 vs. Thy-1 Ab, Thy-1 Ab + HIS, Thy-1 Ab + C6DS, and MEM; ^ΔΔ^p < 0.01 vs. Thy-1 Ab + C6DS). Data are representative of three independent experiments with similar results or are shown as mean ± SD from three independent experiments. Thy-1N, Thy-1 nephritis; GMCs, glomerular mesangial cells; IB, immunoblotting; NS, normal rabbit serum; MEM, minimum essential medium.

We assessed the expression of KLF4 and IL-6 to determine whether KLF4 may regulate IL-6. Our data showed that in renal tissue of Thy-1N rats (*in vivo*), the mRNA and protein levels of KLF4 and IL-6 were elevated in a time-dependent manner with a peak at 3 h (**Figures 1A–D**). Besides, in GMCs stimulated with sublytic C5b-9 (*in vitro*), the mRNA and protein levels of KLF4 and IL-6 were also upregulated in a time-dependent manner and reached their peak at 3 h (**Figures 1E, F**).

Subsequently, to make sure that the increase of KLF4 and IL-6 is actually due to assembly of sublytic C5b-9 complex, GMCs were treated with sublytic C5b-9, Thy-1 Ab, Thy-1 Ab + HIS, Thy-1Ab + C6DS, and MEM for 3 h. The results exhibited that the expression of KLF4 and IL-6 was significantly upregulated in the sublytic C5b-9 group (**Figures 1G, H**). Moreover, adding C6 back to Thy-1 Ab + C6DS treatment recovered the ability to induce KLF4 and IL-6 syntheses (**Figures 1G, H**), confirming that the expression of KLF4 and IL-6 is indeed triggered by sublytic C5b-9. Collectively, these data demonstrated that KLF4 was almost simultaneously expressed with IL-6, indicating that KLF4 may relate to IL-6 production in sublytic C5b-9-induced GMCs.

### KLF4 Promotes IL-6 Expression by Modulating Its Transcription

**Figure 2:**
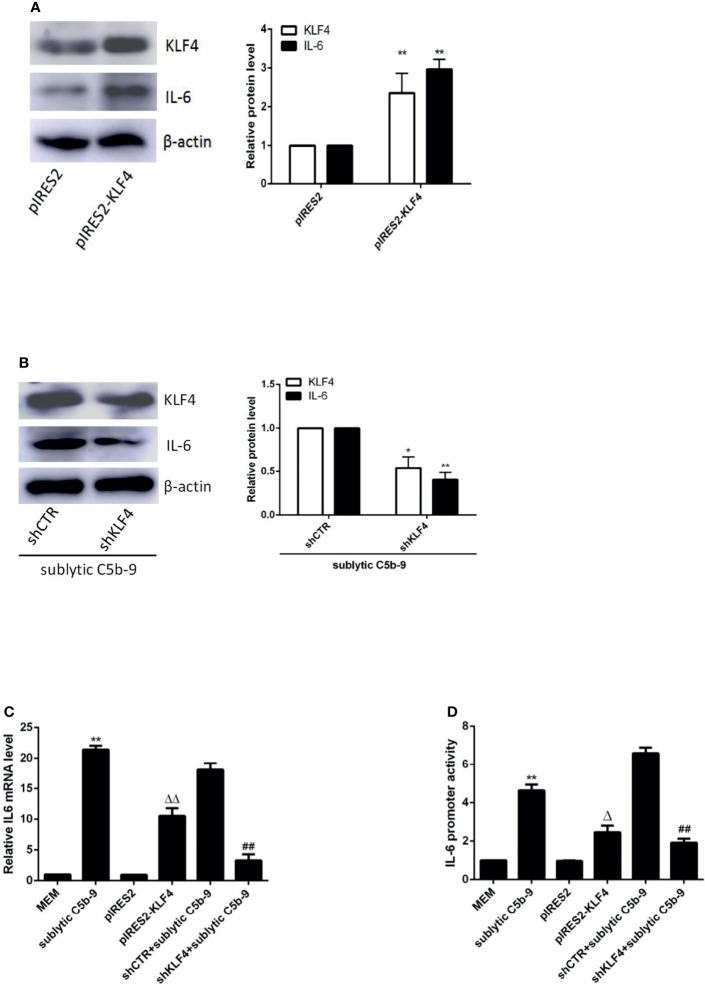
Effect of KLF4 on IL-6 expression in sublytic C5b-9-stimulated GMCs. **(A–C)** GMCs were transfected with pIRES2, pIRES2-KLF4, shCTR, or shKLF4 vector for 48 h and then incubated with or without sublytic C5b-9 for 3 h. **(A, B)** IB analysis of KLF4 and IL-6 (*p < 0.05, **p < 0.01 vs. pIRES2 or shCTR + sublytic C5b-9). **(C)** qPCR analysis of IL-6 (**p < 0.01 vs. sublytic C5b-9; ^ΔΔ^p < 0.01 vs. pIRES2; ^##^p < 0.01 vs. shCTR + sublytic C5b-9). **(D)** Luciferase activity assay of IL-6 reporter (−1,791 to +30 nt) (i.e., full-length construct) in GMCs transfected with pIRES2, pIRES2-KLF4, shCTR, or shKLF4 for 48 h and then stimulated with or without sublytic C5b-9 for 3 h (**p < 0.01 vs. sublytic C5b-9; ^Δ^p < 0.01 vs. pIRES2; ^##^p < 0.01 vs. shCTR + sublytic C5b-9). Data are representative of three independent experiments with similar results or are shown as mean ± SD from three independent experiments. GMCs, glomerular mesangial cells; IB, immunoblotting.

In order to elucidate whether KLF4 is necessary to induce IL-6 production, overexpression and knockdown approaches were used. As shown in **Figures 2A–C**, forced exogenous KLF4 expression markedly increased the mRNA and protein levels of IL-6, whereas interference with endogenous KLF4 expression notably decreased IL-6 production in GMCs, suggesting that KLF4 is required for IL-6 expression.

To determine whether KLF4 can regulate IL-6 at the transcriptional level, luciferase reporter assays of rat IL-6 proximal promoter were performed. In comparison with MEM treatment, the activity of the IL-6 promoter was greatly enhanced upon sublytic C5b-9 stimulation in GMCs (**Figure 2D**). Importantly, KLF4 overexpression boosted activation of the IL-6 promoter, whereas KLF4 knockdown suppressed activation of the IL-6 promoter (**Figure 2D**), implicating that KLF4 functions as a positive regulator of IL-6 gene transcription in GMCs exposed to sublytic C5b-9.

### KLF4 Activates IL-6 Promoter by Binding to Proximal Binding Site

**Figure 3:**
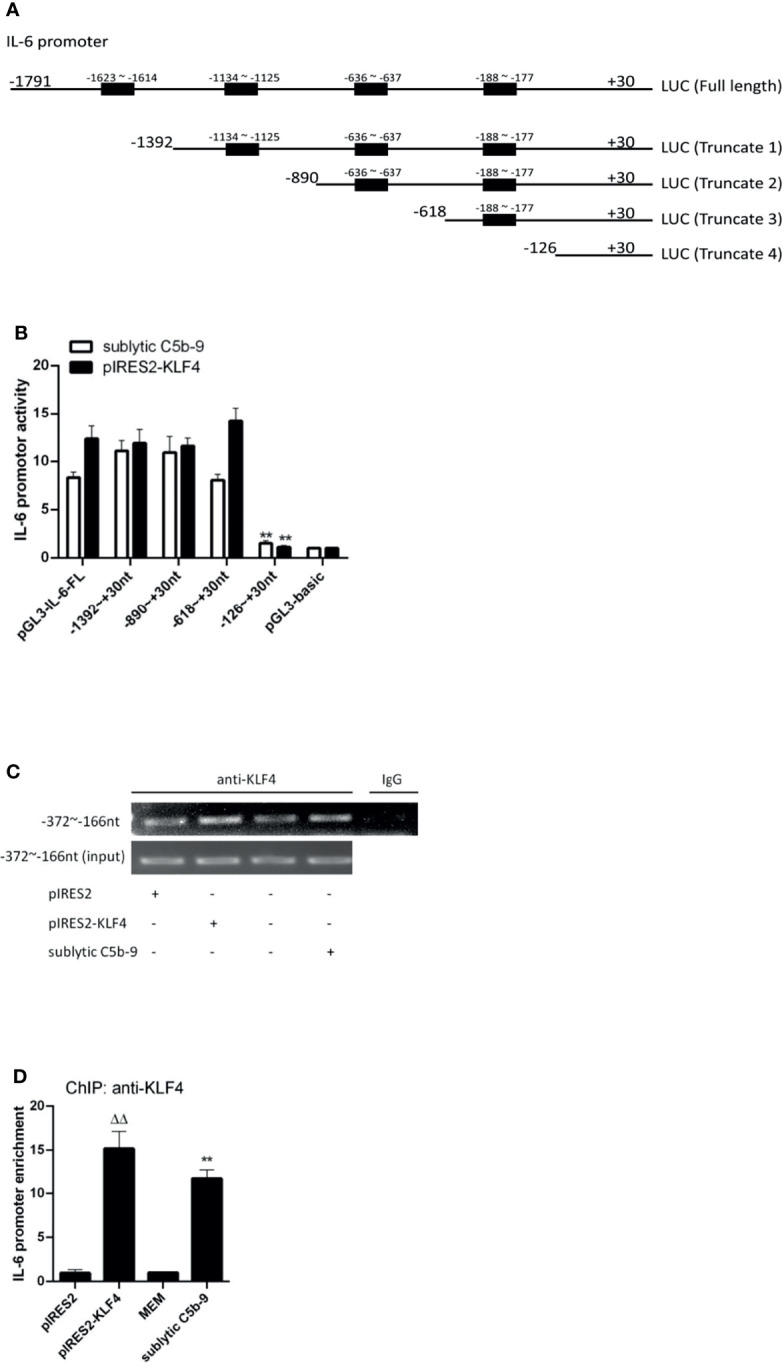
Identification of KLF4 binding site on rat IL-6 gene promoter. **(A)** Schematic representation of IL-6 promoter deletion mutants used. **(B)** Luciferase activity assay of IL-6 reporter in GMCs transfected with full length (FL) or different truncation mutants of IL-6 reporter accompanied by sublytic C5b-9 stimulation for 3 h or together with pIRES2-KLF4 transfection for 48 h (**p < 0.01 vs. pGL3-IL-6-FL). **(C, D)** ChIP–RT-PCR **(C)** and ChIP–qPCR **(D)** analyses of KLF4 enrichment on IL-6 promoter (−372 to −166 nt) in GMCs transfected with pIRES2, or pIRES2-KLF4 for 48 h and then incubated with sublytic C5b-9 or without sublytic C5b-9 (i.e., MEM control) for 3 h (**p < 0.01 vs. MEM; ^ΔΔ^p < 0.01 vs. pIRES2). Data are representative of three independent experiments with similar results or are shown as mean ± SD from three independent experiments. GMCs, glomerular mesangial cells; ChIP, chromatin immunoprecipitation; MEM, minimum essential medium.

The FL IL-6 promoter (−1,791 to +30 nt) contained 4 putative KLF4 binding sites detected in JARSPAR database as shown in **Figure 3A**. To assess whether KLF4 can modulate IL-6 promoter activity by directly binding to a particular site, we performed luciferase reporter assays with different IL-6 promoter deletion mutants. As shown in **Figure 3B**, although the FL and 3 truncation mutant (−1,392 to +30, −890 to +30, and −618 to +30 nt) constructions exhibited significant IL-6 promoter activity, only the shortest truncation mutant (−126 to +30 nt) presented notably attenuated IL-6 promoter activity upon sublytic C5b-9 stimulation or KLF4 overexpression, indicating that the proximal sequences (−618 to −126 nt) likely harbor an effective KLF4 binding site.

Because a putative KLF4 binding site (−188 to −177 nt) belongs to sequences (−618 to −126 nt), we next sought to verify that KLF4 binds to this region by using ChIP assays. The enrichment of KLF4 on the IL-6 promoter region (−372 to −166 nt) was greatly increased after sublytic C5b-9 stimulation or KLF4 overexpression (**Figures 3C, D**). Taken together, these results hint that activation of the IL-6 promoter by KLF4 has resulted from the binding to the proximal promoter region (−372 to −166 nt).

### KLF4 Expression Is Not Regulated by NF-κB Signaling Pathway

**Figure 4:**
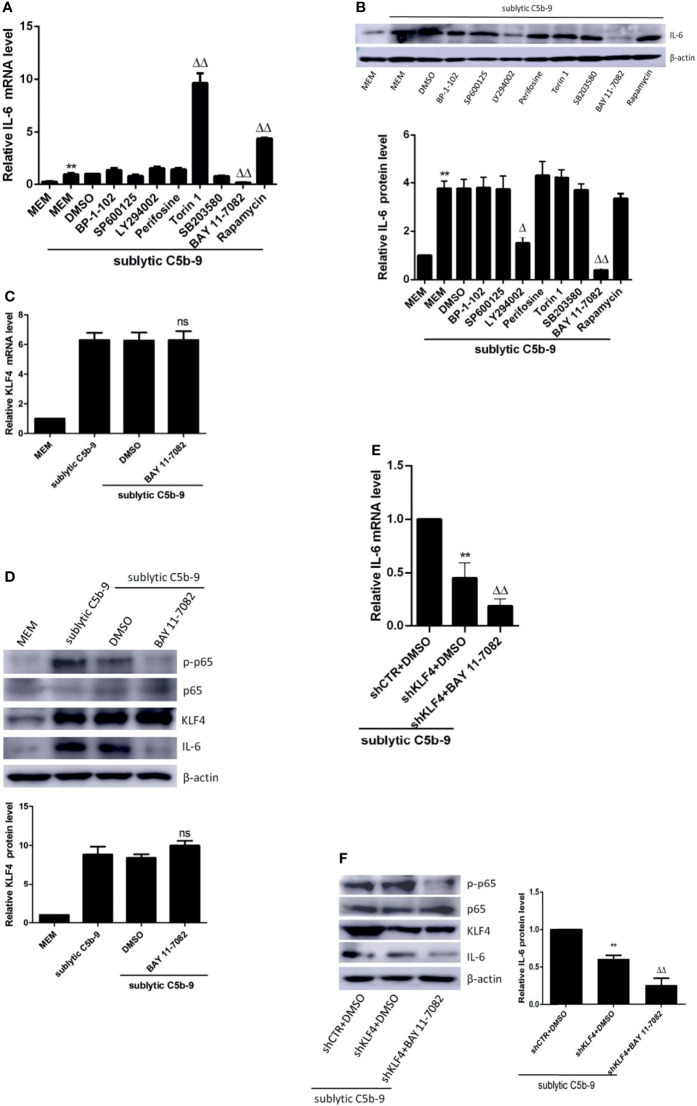
Effect of NF-κB signaling pathway on KLF4 expression. **(A, B)** GMCs were pretreated with inhibitors of BP-1-102, SP600125, LY294002, perifosine, Torin 1, SB203580, BAY 11-7082, or rapamycin at the same dose (10 μM) for 30 min and then stimulated with sublytic C5b-9 for 3 h. qPCR **(A)** and IB **(B)** analyses of IL-6 (**p < 0.01 vs. MEM; ^Δ^p < 0.05, ^ΔΔ^p < 0.01 vs. DMSO + sublytic C5b-9). **(C, D)** GMCs were pretreated with BAY 11-7082 for 30 min and then stimulated with sublytic C5b-9 for 3 h. qPCR **(C)** and IB **(D)** analyses of KLF4, p-NF-κB p65, NF-κB p65, or IL-6 (ns represents p > 0.05 vs. DMSO + sublytic C5b-9). **(E, F)** GMCs were transfected with shCTR or shKLF4 plus DMSO or BAY 11-7082 treatment followed by sublytic C5b-9 attract for 3 h. qPCR **(E)** and IB **(F)** analyses of IL-6, p-NF-κB p65, NF-κB p65, or KLF4 (*p < 0.05, **p < 0.01 vs. shCTR + DMSO + sublytic C5b-9; ^ΔΔ^p < 0.01 vs. shKLF4 + DMSO + sublytic C5b-9). Data are representative of three independent experiments with similar results or are shown as mean ± SD from three independent experiments. GMCs, glomerular mesangial cells; IB, immunoblotting; MEM, minimum essential medium; DMSO, dimethyl sulfoxide.

Given that KLF4 expression is regulated at the transcriptional level (18), we next investigated which signaling pathway may play a role in the regulation of KLF4 expression and KLF4-mediated transactivation. We have previously exhibited that sublytic C5b-9 activates several signaling pathways, including p38 MAPK (29), JNK (29), and PI3k/Akt (30) pathways, in GMCs. There are also evidences to suggest that NF-κB (31), STAT3 (32), and mTOR (33) are activated in Thy-1N model. Accordingly, we first explored which of these pathways may be involved in IL-6 expression by sublytic C5b-9 stimulus. To this end, GMCs were pretreated with the corresponding inhibitors before sublytic C5b-9 attack. SB203580 is an inhibitor of p38 MAPK activity, SP600125 is a JNK inhibitor, LY294002 is a PI3k inhibitor, perifosine is an Akt inhibitor, BAY 11-7082 is an NF-κB inhibitor, BP-1-102 is a STAT3 inhibitor, Torin 1 inhibits mTORC1 and mTORC2, and rapamycin is an inhibitor of mTORC1. The results displayed that the mRNA and protein levels of IL-6 were remarkably decreased by BAY 11-7082 (**Figures 4A, B**), implying that NF-κB activation promotes IL-6 production in GMCs stimulated with sublytic C5b-9.

Then, we sought to assess whether NF-κB activation can regulate the expression of KLF4. **Figures 4C, D** illustrate that BAY 11-7082, which decreased the phosphorylation level of p65, did affect neither KLF4 mRNA level nor KLF4 protein level, hinting that KLF4 expression is not modulated by NF-κB pathway in sublytic C5b-9-treated GMCs. Moreover, treatment with BAY 11-7082 in shKLF4-transfected GMCs led to a stronger decrease in IL-6 expression at both the mRNA and protein levels (**Figures 4E, F**). These results altogether suggest that NF-κB activation promotes IL-6 expression, which is not *via* induction of KLF4 expression in sublytic C5b-9-stimulated GMCs.

### KLF4 Acetylation by PCAF Encourages IL-6 Transcription

**Figure 5:**
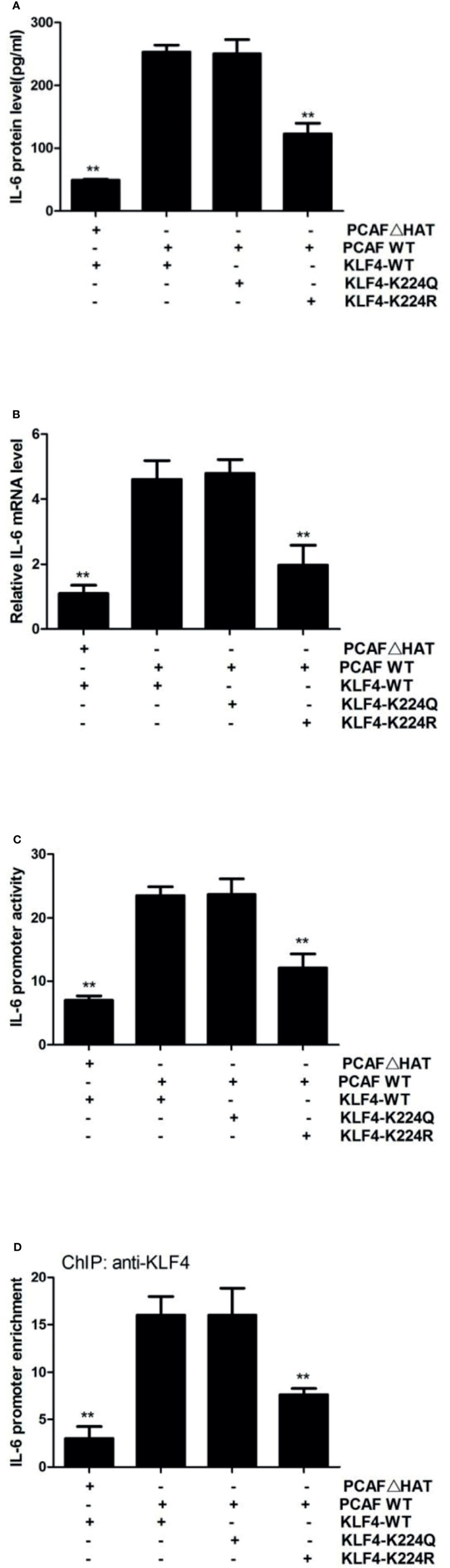
Effect of KLF4 acetylation by PCAF on IL-6 gene transcription in GMCs exposed to sublytic C5b-9. GMCs were transfected with wild-type PCAF (PCAF WT), PCAF HAT-deficient mutant (PCAFΔHAT), wild-type KLF4 (KLF4 WT), acetylation-deficient mutant (KLF4-K224R), or hyper-acetylated mutant (KLF4-K224Q) for 48 h. **(A)** ELISA of IL-6 in supernatants of GMCs. **(B)** qPCR analysis of IL-6. **(C)** Luciferase activity assay of IL-6 reporter (−1,791 to +30 nt). **(D)** ChIP–qPCR analysis of KLF4 enrichment on IL-6 promoter (−372 to −166 nt). **p < 0.01 vs. PCAF WT + KLF4 WT. Data are shown as mean ± SD from three independent experiments. GMCs, glomerular mesangial cells; ChIP, chromatin immunoprecipitation.

KLF4-mediated transactivation is also regulated at the posttranslational level (18). We previously have found that KLF4 is acetylated by acetyltransferase PCAF and that mutant of KLF4 at lysine residue 224 results in a decreased ability of KLF4 to active IL-23 and IL-36 in sublytic C5b-9-stimulated GMCs (9). Thus, we decided to reveal whether lysine 224 of KLF4 is important for IL-6 induction as well. We found that compared with overexpression of wild-type KLF4, overexpression of acetylation-deficient mutant KLF4-K224R resulted in a lessened IL-6 expression, whereas overexpression of hyper-acetylated mutant KLF4-K224Q caused almost no change of IL-6 expression (**Figures 5A, B**). Similarly, KLF4-K224R led to a notable decrease in IL-6 promoter activation, but KLF4-K224Q produced almost no change (**Figure 5C**). Additionally, the IL-6 promoter was less occupied by KLF4 when using K224R mutant, whereas the level of KLF4 occupancy at the IL-6 promoter was to the same extent with wild-type KLF4 when using K224Q mutant (**Figure 5D**). Furthermore, overexpression of the HAT-deficient PCAF mutant resulted in a significant decrease of IL-6 expression, IL-6 promoter activity, and KLF4 occupancy at the IL-6 promoter, compared with overexpression of the wild-type PCAF (**Figures 5A–D**). Overall, these findings strongly denote that PCAF-mediated KLF4 acetylation can regulate IL-6 transcription.

### Histone Acetylation by PCAF Modulates IL-6 Transcription

**Figure 6:**
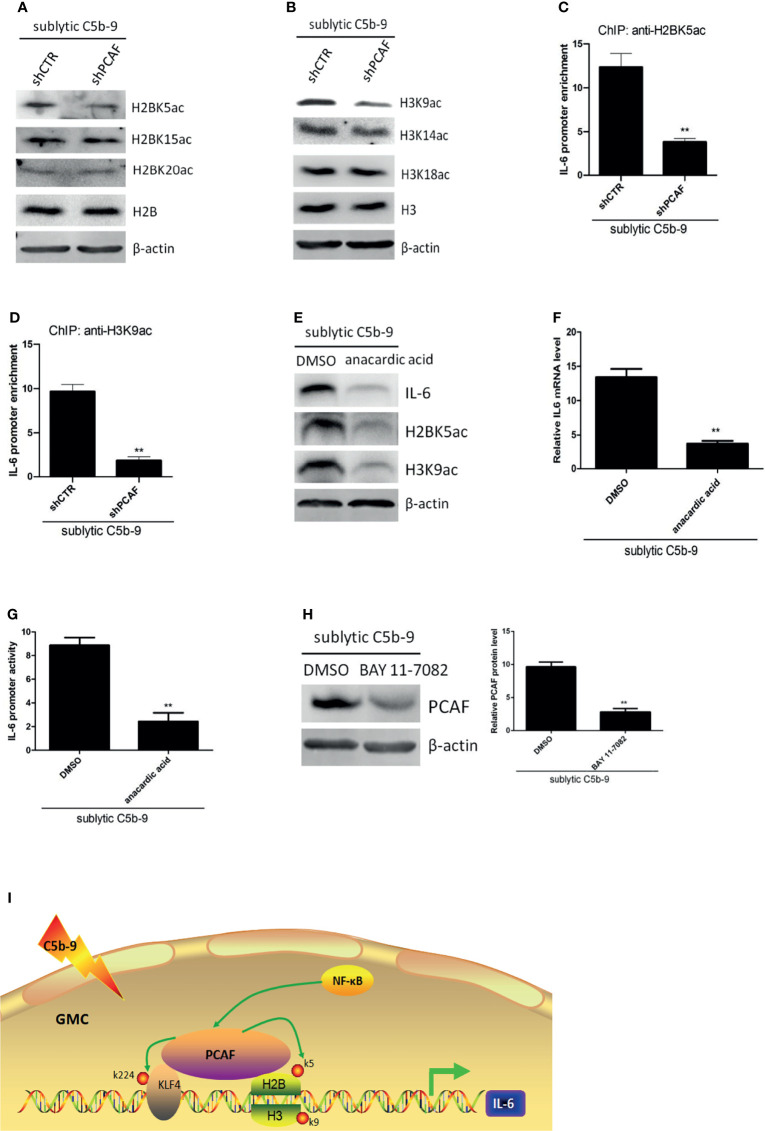
Effect of histone acetylation by PCAF on IL-6 gene transcription in GMCs in response to sublytic C5b-9. **(A–D)** GMCs were transfected with shCTR or shPCAF for 48 h and then incubated with sublytic C5b-9 for 3 h. **(A)** IB analysis of H2B acetyl K5, H2B acetyl K15, H2B acetyl K20, and H2B. **(B)** IB analysis of H3 acetyl K9, H3 acetyl K14, H3 acetyl K18, and H3. **(C)** ChIP–qPCR analysis of enrichment of H2B acetyl K15 on IL-6 promoter (−372 to −166 nt). **(D)** ChIP–qPCR analysis of enrichment of H3 acetyl K9 on IL-6 promoter (−372 to −166 nt). **p < 0.01 vs. shCTR + sublytic C5b-9. **(E–G)** GMCs were pretreated with DMSO or anacardic acid (30 μM) for 1 h and then stimulated with sublytic C5b-9 for 3 h. **(E)** IB analysis of IL-6, H2B acetyl K5, and H3 acetyl K9. **(F)** qPCR analysis of IL-6. **(G)** Luciferase activity assay of IL-6 reporter (−1,791 to +30 nt). **p < 0.01 vs. DMSO + sublytic C5b-9. **(H)** GMCs were pretreated with BAY 11-7082 for 30 min and then stimulated with sublytic C5b-9 for 3 h. IB analysis of KLF4 (**p < 0.01 vs. DMSO + sublytic C5b-9). **(I)** A putative scheme for regulatory mechanisms of IL-6 expression induced by sublytic C5b-9 in GMCs. KLF4 binds to IL-6 promoter and activates its transcription. PCAF acetylates KLF4 (at K224) and neighboring histones H2B (at K5) and H3 (at K9), contributing to IL-6 transcription. Besides, NF-κB signaling pathway also regulates IL-6 expression *via* increasing PCAF expression. Data are representative of three independent experiments with similar results or are shown as mean ± SD from three independent experiments. GMCs, glomerular mesangial cells; IB, immunoblotting; DMSO, dimethyl sulfoxide.

Inhibition of PCAF by the HAT-deficient mutant significantly blocked KLF4-mediated transcriptional activation (**Figure 5C**). However, KLF4-K224R mutation only partially inhibited this activity (**Figure 5C**), suggesting that PCAF-mediated acetylation activates IL-6 gene by more than one mechanism. Since a major function of PCAF is to acetylate histones (26) and histone acetylation has been linked to gene transcription for decades (34), we hypothesized that PCAF-mediated histone acetylation may contribute to IL-6 activation. Toward this end, we first analyzed the effect of PCAF on histone acetylation status. By using antibodies that detected different specific histone acetylation sites, we found that the levels of H2BK5ac and H3K9ac were significantly downregulated in shPCAF-pretreated GMCs, whereas the levels of H2BK15ac, H2BK20ac, H3K14ac, and H3K18ac were similar between shPCAF-pretreated and shCTR-pretreated GMCs (**Figures 6A, B**), revealing that K5 on histone H2B and K9 on histone H3 are catalyzed by PCAF in sublytic C5b-9-induced GMCs. Because the majority of histone acetylation is limited to the promoter of genes (35), we next sought to test whether the PCAF-manipulated H2BK5ac and H3K9ac have occurred at the IL-6 promoter. ChIP assays were performed by using primers that were designed to target the KLF4 binding site (−372 to −166 nt) on the IL-6 promoter. As shown in **Figures 6C, D**, PCAF knockdown induced significant decreases of H2BK5ac and H3K9ac recruitment on the IL-6 promoter in GMCs.

To further ascertain the effect of histone acetylation on IL-6 activation, sublytic C5b-9-attacked GMCs were pretreated with anacardic acid, which is a pan-histone acetylase inhibitor. We observed that the mRNA and protein levels of IL-6 were reduced in the presence of anacardic acid (**Figures 6E, F**). Luciferase reporter assays proved that IL-6 promoter activity was decreased in the presence of anacardic acid (**Figure 6G**). Collectively, these data indicate that PCAF-mediated histone acetylation on the IL-6 promoter region plays a crucial role in the regulation of IL-6 expression, including H2BK5ac and H3K9ac.

Although NF-κB activation regulating IL-6 expression was not *via* the induction of KLF4 expression, we further considered whether it was *via* the induction of PCAF expression. **Figure 6H** shows that PCAF protein level was decreased by NF-κB activity blockade.

## Discussion

Inflammatory molecules play important pathogenic roles both in rat Thy-1N and in human MsPGN (8–10, 36). Elucidation of the molecular mechanisms by which cytokine production is regulated is a critical step toward deciphering the inflammatory injury. We have found that IL-6 is obviously upregulated both in the renal tissues of Thy-1N rats (*in vivo*) and in GMCs exposed to sublytic C5b-9 (*in vitro*) (8), but the molecular mechanisms regulating its expression have not been completely understood.

Control of IL-6 expression is multifactorial, and a number of transcription factors have been validated to participate in its regulation (8, 15, 16). Our published work has demonstrated that C/EBPβ is involved in regulating IL-6 expression of Thy-1N rats through its binding to the IL-6 promoter at sequences (−618 to −126 nt) in GMCs subjected to sublytic C5b-9 attack (8). In this study, we further analyzed this promoter region by using bioinformatics database and found that it also harbored potential binding elements for transcription factor KLF4. Importantly, we found that the raised expression of KLF4 was highly synchronous with that of IL-6 both *in vivo* and *in vitro*. Thus, we hypothesized that KLF4 may modulate IL-6 production. The data showed that the expression of IL-6 was significantly increased by enforcing overexpression of KLF4 in GMCs, while that of IL-6 was markedly suppressed by silencing KLF4 in sublytic C5b-9-induced GMCs. These findings indicate that KLF4 indeed regulates IL-6 expression in GMCs of Thy-1N rats.

Because of the known role as a transcription factor of KLF4, we sought to systematically determine how it interacts with the IL-6 promoter. KLF4 has been reported to modulate the activation of various inflammatory mediators *via* binding to GC box or CACCC element on target genes (18–20). In this study, multiple possible binding sites for KLF4 on rat the IL-6 promoter were detected by using JARSPAR database. Luciferase reporter assays, which were performed with systematic dissection of the different potential binding sites, and ChIP assays substantiated that KLF4 mediated its primary transcriptional effect on the IL-6 promoter through binding to sequences (−372 to −166 nt). This proximal promoter region contained the CACCC motif (−188 to −177 nt), which may be the real element for KLF4 on rat IL-6 promoter activity. These results suggest that KLF4 activates the transcription of IL-6 in sublytic C5b-9-induced GMCs by directly binding to the IL-6 promoter.

Interestingly, the binding site for C/EBPβ (8) is close to the binding site for KLF4 on rat IL-6 proximal promoter in GMCs. Given that KLF4 has been proved to interact with other transcription factors (NANOG, SOX2, and OCT4) in order to regulate genes (37), we consider that KLF4 may interact with C/EBPβ upon sublytic C5b-9 stimulation, and this interaction may be a benefit for transactivation of IL-6. However, this hypothesis requires further research.

The expression of KLF4 is regulated at the transcriptional level. It has been reported that multiple signaling pathways regulate the expression pattern of KLF4 *via* their effectors (18). In this study, we used different signaling pathway inhibitors and made the observation that NF-κB activation was involved in IL-6 expression in sublytic C5b-9-treated GMCs, but it was not involved *via* modulation of KLF4 expression. Unexpectedly, NF-κB promoting IL-6 expression could through increasing PCAF protein.

The activity of KLF4 is regulated at the posttranslational level. Several researchers have pointed out that KLF4-mediated gene transcription is affected on multiple levels by modulating the status of KLF4 through phosphorylation, acetylation, methylation, and ubiquitination in a context-dependent manner (9, 38–40). In sublytic C5b-9 condition, KLF4 has previously been reported to regulate the transcription of IL-23 and IL-36a *via* acetylation by PCAF (9). By using HAT-deficient mutant of PCAF and site-directed mutagenesis of KLF4, our current study demonstrated that acetylation of KLF4 at lysine residue 224 (K224) by PACF could also promote IL-6 transcription. This study supports the notion that acetylation modification is crucial for transcription factor-dependent transactivation (27).

In general, acetylation may modulate a number of functional properties of a transcription factor, including its sequence-specific DNA binding activity, the affinity of interaction with other transcriptional regulators, and the proper nuclear retention, preventing its active export to the cytoplasm (28). Among these, the DNA-binding property of KLF4 was investigated in this study. We found that overexpression of KLF4 together with PCAF HAT-deficient mutant exhibited reduced KLF4 occupancy at the IL-6 promoter. However, the possibility that acetylation may influence other properties of KLF4 needs to be confirmed in further experiments.

In addition to several transcription factors, histone acetylation at the IL-6 promoter has also been linked to IL-6 transcription (19). PCAF, which possesses an intrinsic HAT activity, is capable of acetylating histones as well (26). In the current study, our data showed that K5 on histone H2B and K9 on histone H3 at the IL-6 promoter were catalyzed by PCAF and that histone acetylation modulated the transcription of IL-6. These data indicate that PCAF-mediated histone acetylation, including H2BK5ac and H3K9ac, on the IL-6 promoter regulates IL-6 expression in sublytic C5b-9-induced GMCs. The deep reason for this has been uncovered that histone acetylation on lysines neutralizes the charge on histones, which weakens the binding between histones and DNA, making transcription factors and the basal transcriptional machinery more accessible for DNA and then facilitating gene transcription (41).

Usually, the level of histone acetylation is dynamically controlled by the activity of HATs and histone deacetylases (HDACs). The possible HDAC inhibition involved in IL-6 expression by GMCs upon sublytic C5b-9 stimulation needs further investigation.

In summary, our study revealed molecular mechanisms underlying the production of IL-6 induced by sublytic C5b-9 in rat GMCs. We propose a model (**Figures 6I**) that KLF4 binds to proximal sequences (−618 to −126 nt) on the IL-6 promoter and activates IL-6 gene transcription. Then, KLF4 recruits PCAF to the IL-6 promoter. PCAF acetylates KLF4 at K224 and promotes IL-6 transcription *via* an increase of KLF4 occupancy at this IL-6 proximal promoter sequences. Meanwhile, PCAF also acetylates histone H2BK5 and histone H3K9 at the same IL-6 proximal promoter sequences, resulting in an enhancement of IL-6 transcription. Additionally, NF-κB activation also regulates IL-6 expression, and this process is through elevating PCAF protein.

## Data Availability Statement

The original contributions presented in the study are included in the article/supplementary material. Further inquiries can be directed to the corresponding author.

## Ethics Statement

The animal study was reviewed and approved by the Institutional Animal Care and Use Ethics Committee of Nanjing Medical University.

## Author Contributions

LX, YL, ZZ, YG, and TY performed the experiments. LX, DZ, and WQ analyzed the data. JZ wrote the manuscript. JZ and YW designed and supervised the study. All authors contributed to the article and approved the submitted version.

## Funding

This work was supported by grants from the National Natural Science Foundation of China (31500701 and 81971468).

## Conflict of Interest

The authors declare that the research was conducted in the absence of any commercial or financial relationships that could be construed as a potential conflict of interest.

## Publisher’s Note

All claims expressed in this article are solely those of the authors and do not necessarily represent those of their affiliated organizations, or those of the publisher, the editors and the reviewers. Any product that may be evaluated in this article, or claim that may be made by its manufacturer, is not guaranteed or endorsed by the publisher.
